# Abomasitis associated with halofuginone intoxication in pre-weaned calves

**DOI:** 10.1186/s12917-023-03850-7

**Published:** 2024-01-03

**Authors:** Wouter van Mol, Laurens Chantillon, Lise Geerinckx, Jolien Coppens, Justine Clinquart, Mathilde Pas, Jade Bokma, Bart Pardon

**Affiliations:** 1 Department of Internal Medicine, Reproduction and Population Medicine, Faculty of Veterinary Medicine, Ghent University, Salisburylaan 133, Merelbeke, 9820 Belgium; 2 Veterinary Center Trigenio, Dorsel 38, Medvet-AML, Emiel Vloorsstraat 9, 2560, 2020 Nijlen, Antwerp, Belgium

**Keywords:** Halofuginon lactate, Intoxication, Abomasitis, Ultrasonography

## Abstract

**Background:**

In this case series abomasitis as a consequence of halofuginone intoxication is suspected.

**Case presentation:**

Seven Belgian-Blue calves with complaints of anorexia and weight loss were presented to an university clinic. Ultrasonography showed thickening and edema of the abomasal wall in all cases, suggesting abomasitis. Abomasitis was confirmed on necropsy in three cases. Retrospective analysis clarified the uptake of an overdose of halofuginone lactate (348–421 µg/kg/day). Four animals fully recovered after removal of halofuginone lactate administration, therapy for comorbidities (pneumonia, diarrhoea) and supportive therapy.

**Conclusion:**

To the authors’ knowledge, this case series is the first report associating halofuginone lactate use with abomasitis. This was suspected after clinical improvement of four of the presented animals after terminating the administration of a high dose of halofuginone lactate, and exclusion of other possible causes. Underlying mechanisms are still unclear.

## Background

Preventive treatment with halofuginone lactate is common practice against cryptosporidiosis, one of the most prevalent causes of neonatal calf diarrhoea worldwide [1]. Timely administration, before the age of five days, showed to decrease oocyst shedding, severity of diarrhoea and mortality after infection [2–5]. In order to prevent severe clinical signs, the recommended effective dose is between 100 and 120 µg/kg per day [6]. The use of halofuginone lactate as a curative treatment is not recommended, as it cannot be given to weakened or dehydrated calves, or those showing clinical signs for more than 24 h [7]. Furthermore, evidence for the therapeutic usage of halofuginone lactate is insufficient [8]. An additional limiting property for the use of halofuginone lactate is its toxicity. Diarrhoea, blood in faeces, anorexia, dehydration and prostration have been reported at daily doses of ≥ 200 µg/kg [7]. The following short report describes a case series of abomasitis, associated with overdosing of halofuginone lactate in calves.

## Case presentation

Between the end of March and beginning of April 2021, seven Belgian-Blue beef calves were presented at the clinic of Large Animal Internal Medicine of Ghent University (Belgium) with complaints of anorexia and subsequent weight loss. The animals originated from a closed Belgian Blue beef farm (approximately 100 animals). All calves are individually kept on straw in igloos after birth. The farm has an all year calving pattern, although the calving density can vary throughout the year. Therefore, individual housing length depends on the supply of calves (maximum of eight weeks).

The farmer stated that this problem had been going on for multiple weeks and usually started around one week of age. Some calves developed additional diarrhoea and/or pneumonia. Three calves with clinical signs of pneumonia had already died. No necropsy was performed on these calves. In addition, the farm had ongoing problems with neonatal calf diarrhoea for years. No infectious diagnostics were performed so far. Due to these on-farm problems with diarrhoea, the farmer prophylactically administered halofuginone lactate (Halocur, Intervet International, Boxmeer, NL) at the dose ranging from 174 µg/kg to 211 µg/kg in the milk to all calves starting from 2 days of age. However, as a response to the recent anorexia and weight loss problems, the daily given dose was increased to dosings ranging from 348 µg/kg tot 421 µg/kg for all calves up to one month of age. These changes were made without consultation of a veterinarian.

On farm, multiple animals did receive therapeutic treatment upon decline of the appetite and after visitation by the veterinarian with parental administration of 15 mg/kg amoxicillin (Vetrimoxin Long Acting, Ceva Santé Animale NV/SA, Brussels, Belgium) or 10 mg/kg lincomycin with 5 mg/kg spectinomycin (Emdactilin, Emdoka bv, Hoogstraten, Belgium) for the duration of three to four days, respectively, and a one-time intramuscular administration of 0.5 mg/kg meloxicam (Metacam, Boehringer Ingelheim, Ingelheim am Rhein, Germany) without any apparent effect. The weight of the animals was estimated by the veterinarian. The animals received standard 1,5–2 L of milk replacer (382 mOsm/kg) at the recommended concentration of 150 g/L (Spraystart E, Aveve, Leuven, Belgium), four times a day.

Upon arrival in the clinic, a clinical examination was performed on all seven calves together with blood examination and thoracic and abdominal ultrasound. A detailed summary of the demographic characterisations and clinical examination is given in Table 1. All animals had weight loss, as they weighed less than the average birth weight of a Belgian Blue, which is approximately 50 kg [9], and showed signs of dehydration. Four animals did not produce any faeces, while two others had diarrhoea.

**Table 1:** Summary of clinical examination of seven calves with halofuginone intoxication. Reference values are based on Rebhun’s Diseases of Dairy Cattle [10]

	Calf 1	Calf 2	Calf 3	Calf 4	Calf 5	Calf 6	Calf 7	Reference*
**Age**	29 d	28 d	28 d	4 d	4 d	26 d	4 d	
**Weight**	46 kg	39 kg	38 kg	45 kg	40 kg	35 kg	45 kg	
**Body temperature**	38.7 °C	38.6 °C	39.0 °C	38.6 °C	39.7 °C	38.8 °C	38.3 °C	38–39.5 °C
**Heart frequency**	108/min	72/min	148/min	88/min	112/min	96/min	60/min	72–100/min
**Breathing frequency**	28/min	24/min	36/min	44/min	56/min	28/min	36/min	
**Oral mucosa**	Hyperemia	Pink	Hyperemia	Hyperemia	Hyperemia	Hyperemia	Pink	Pink
**Capillar refill time**	> 2s	> 2s	< 2s	> 2s	> 2s	> 2s	> 2s	< 2s
**Skin pinch**	Delayed	Delayed	Delayed	Delayed	Delayed	Delayed	Delayed	Immediate
**Auscultation thorax**	Increased vesicular	Increased vesicular	Vesicular	Vesicular	Vesicular	Vesicular	Increased vesicular	Vesicular
**Auscultation abdomen**	Left: Fluid splashing	Left: Fluid splashing Right: No sound	No sound	Left: Fluid splashing	Normal	Bilateral fluid splashing	Bilateral fluid splashing	Borborygmes
**Faeces**	Absent	Normal	Absent	Absent	Absent	Liquid	Liquid	Normal

Ultrasonography was performed with a linear 10.5 MHz probe (Sonosite M-Turbo, Fujifilm, Dusseldorf, DE) and 75% isopropanol solution (propanol-2, Chem Lab NV, Zedelgem, Belgium) as transducer agent directly between skin and probe. The thoracic ultrasound showed pneumonia (at least one lung consolidation between 1 and 1.5 cm) in Calf 1, 2, 6 and 7, whereas the abdominal ultrasound showed oedema of the abomasal wall and folds in all animals, signs of enteritis in Calf 7 (oedema of intestinal wall), and free fluid (homogenous anechogenic) near the ventral liver point and between the intestines in Calf 4 and 7.

During clinical examination, blood samples were taken from the vena jugularis with a vacutainer system (Venoject®, Terumo, Leuven, Belgium). Blood-gas analysis (RAPIDPoint® 405, Siemens Healthcare, Beersel, Belgium) showed a metabolic acidosis in Calf 1 and 2, elevated haematocrit in Calf 3, 5, 6 and 7, and a high lactate in Calf 7. Biochemistry analysis (IDEXX Catalyst One Chemistry Analyser®, IDEXX Europe B.V., Hoofddorp, The Netherlands) showed hypoproteinaemia in Calf 1, 2 and 3, elevated blood urea in Calf 2 and 3. An overview of the blood examinations is given in Table 2. In order to reduce costs, total protein was only determined for Calf 1, 2, and 3. Lactate concentration of Calf 1, 2 and 3 could not be determined, due to errors of the blood machine at the time of presentation. Due to the death of Calf 1, 3 and 6 these biochemistry results are incomplete.

**Table 2:** Blood gas analysis and biochemistry results of seven Belgian blue beef calves with halofuginone intoxication at the time of their presentation at the clinic

	Calf 1	Calf 2	Calf 3	Calf 4	Calf 5	Calf 6	Calf 7	References used within clinic
**Haematocrit** (%)	30	35	46	30	44	37	36	**25–35**
**pH**	7.22	7.32	7.34	7.356	7.49	7.383	7.45	**7.35–7.44**
**pCO**_**2**_ (mmHg)	27.7	34.9	34.9	51	41.4	45.2	45.6	**45**
**HCO**_**3**_ (mmol/l)	14.5	17.8	23.3	27.9	30.3	26.2	31.6	**25**
**Base Excess** (meq/l)	-14.8	-7.6	-2.3	1.8	6.8	0.8	6.9	**-5–5**
**Glucose** (mg/dl)	72	90	71	75	106	64	102	**60–100**
**L-Lactate** (mmol/l)	NA	NA	NA	2.33	1.72	7.99	1.94	**< 2**
**Na**^**+**^ (mmol/l)	135	140	138	141.9	141.9	129.3	139.6	**132–152**
**K**^**+**^ (mmol/l)	4.52	4.10	3.93	4.71*	4.97*	4.44	4.30*	**3.50–4.00** * 4.33–5.40
**Ca**^**2+**^ (mmol/l)	1.23	1.22	1.22	1.19	1.16	1.16	1.18	**1.0**
**Cl**^**−**^ (mmol/l)	95	103	101	109	105	94	103	**100**
**Total protein** (g/l)	44	45	52	NA	NA	NA	NA	**62–80**
**Urea** (mmol/l)	NA	9.3	10.4	4.5	4.8	NA	4.5	**3.6–9**
**Creatinine (**µmol/l)	NA	74	NA	141	98	NA	97	**44–141**
**γ-glutamyl-transferase** (IU/l)	NA	20	NA	102	51	NA	47	0–87**
**Aspartate transaminase** (IU/l)	NA	72	NA	84	55	NA	83	**50–100**
**Total bilirubin (**µmol/l)	NA	3	NA	9	6	NA	7	**0–12**

‘NA’ is given when results were not available. All reference values were given by the manufacturer, with the exception of the potassium for calves up to 10 days of age (*). These were derived from Dillane et al. [11]. The serum concentration of γ-glutamyl-transferase can be above reference values up to 40 days of age [12].

All animals were diagnosed with dehydration (skin pinch > 2s) and abomasitis (oedema of the abomasal wall and folds). Some animals showed comorbidities such as pneumonia (Calf 1, 2, 6 and 7) or diarrhoea (Calf 6 and 7). All animals received an off-label proton pump inhibitor in the milk once daily (1 mg/kg Omeprazole Sandoz; Sandoz, Basel (CH)) and intravenous perfusion therapy based on their hydration and acid-base status until resolvement of the clinical signs. Calf 1, 2, and 3 received two intramuscular administrations of 10 mg/kg oxytetracycline (Engemycine 10%, MSD Animal Health, Unterschleissheim, Germany) administered every other day, and Calf 4, 5, 6, and 7 received 22.000 IU/kg procaine benzylpenicillin (Peni-kel, Kela Pharma nv, Hoogstraten, Belgium) for five days. After 4 days of hospitalisation, Calf 2 received 22.000 IE/kg sodium benzylpenicillin (Penicilline 5.000.000 IE, Kela Pharma nv, Hoogstraten, Belgium) four times per day for 8 consecutive days, due to the lack of improvement of the clinical signs.

The animals were fed one litre of milk replacer (Sprayfo Royal, Trouw Nutrition Benelux, Gent, Belgium) at a concentration of 140 g/L, five times a day. This is a standardised procedure within the clinic for the group of youngest milk fed calves. During the first, fourth, and fifth day of hospitalisation, Calf 1, 6, and 3 died, respectively. Full recovery (normal clinical condition, no abnormalities on blood-gas analysis and ultrasonic examination) was obtained by Calf 2, 4, 5, and 7, after 19, 13, 7, and 9 days, respectively. Clinical examination was performed daily and ultrasound every three to five days or at detoriation of clinical status.

**Fig. 1:**
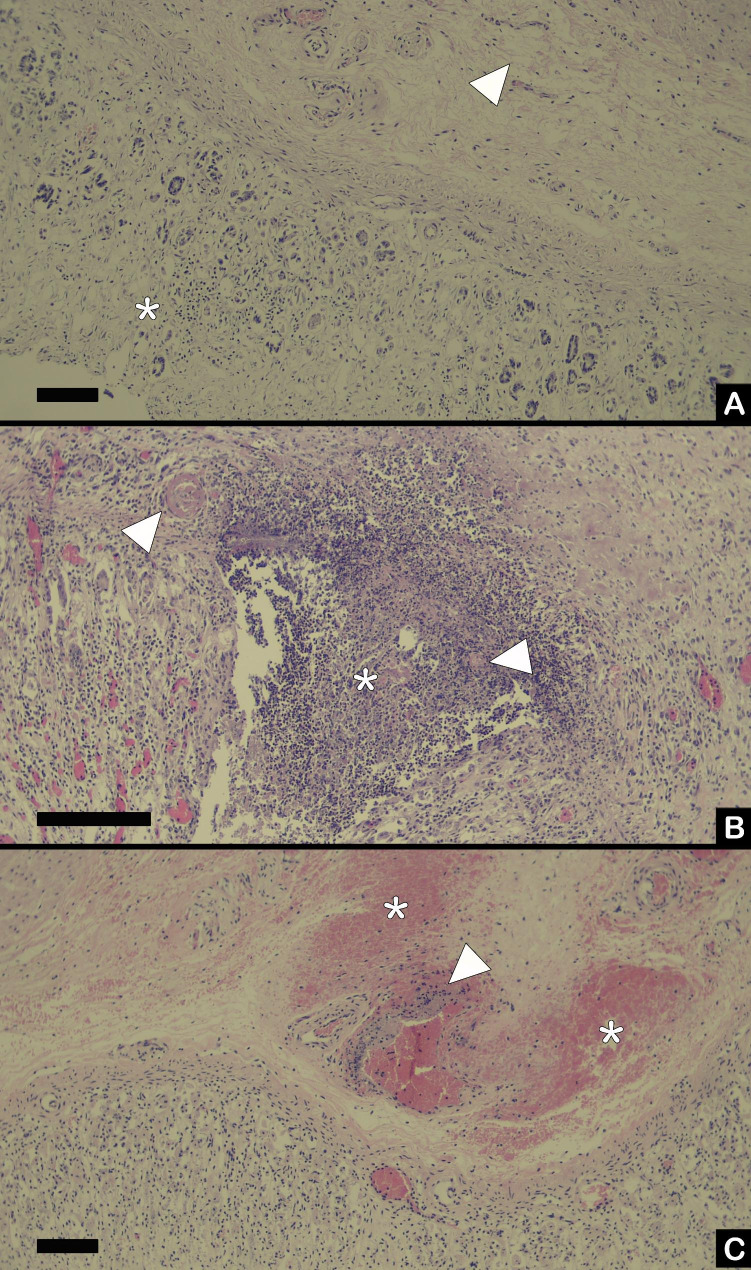
Histology of abomasal lesions of three Belgian-blue beef calves, between 26 and 29 days of age, that died after halofuginone lactate intoxication. All microscopic figures are HE, black bar represents 100 μm. (A) Submucosal edema (arrowhead) and mild lymphoplasmacytic inflammation (asterix) in the mucosa of Calf 1. (B) Submucosal and mucosal vasculitis with presence of thrombi (arrowheads) and necrosis with secondary inflammation (asterix) in Calf 6. (C) Submucosal vasculitis (arrowhead) and diffuse submucosal hemorrhages (asterix) in Calf 3

On necropsy of three calves (Calf 1, 6, and 3), macroscopic and histologic examination was performed. The first calf (Calf 1) showed a dilated abomasum, severely filled with haemorrhagic fluid content mixed with long straw fibres, and a pH of 3 to 6 (pH indicator paper, Carl Roth GmbH, Karslruhe (DE)). The abomasal wall was moderately thickened due to oedema, mucosa had multifocal, poorly delineated, red discolorations, and the serosa had a normal aspect. The rumen, reticulum and omasum showed no significant macroscopic lesions. Other gross lesions in this calf were hepatic pallor and moderate to severe pulmonary oedema. Histologically there was moderate to severe abomasal submucosal oedema and multifocal mild mucosal lymphoplasmacytic infiltration (Fig. 1A.).

**Fig. 2:**
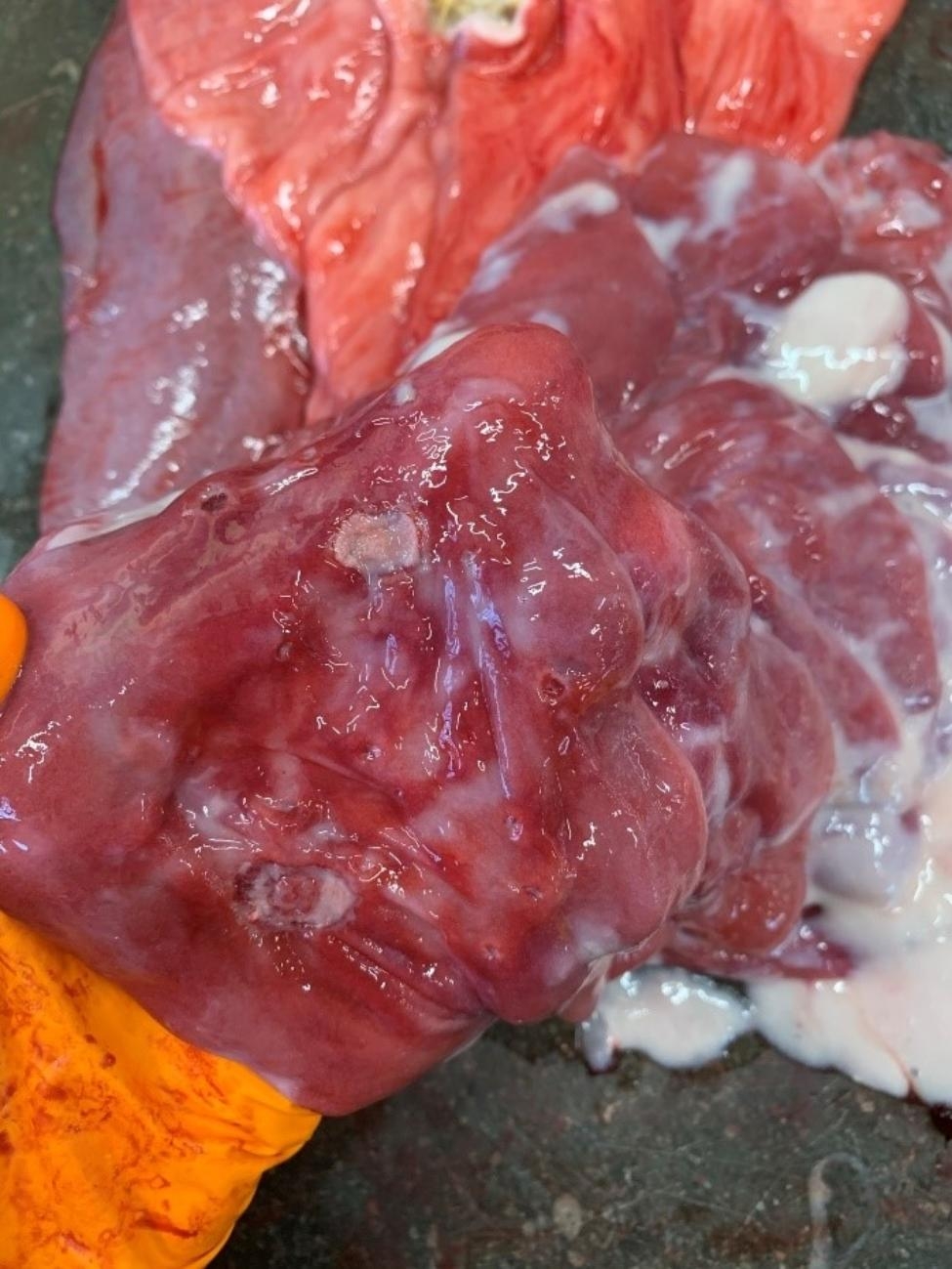
Abomasitis in a Belgian blue beef calf (Calf 6) with halofuginone intoxication, remark the reddened abomsal mucosae with ulcers

The abomasum of Calf 6 had a pH of 5 (pH indicator paper, Carl Roth GmbH, Karslruhe (DE)), and was moderately filled with milk rennet and a small amount of straw. The wall was severely oedematous, mucosa showed a diffuse dark red discoloration with multifocal, sharply delineated, irregular indentations with varying sizes (ulcerations), and serosa was normal (Fig. 2.). Rumen, reticulum and omasum showed no significant abnormalities. Other gross lesions in this calf were cachexia, dehydration, diarrhoea, hepatic pallor, mild cranioventral suppurative bronchopneumonia, and multifocal cerebral and cerebellar meningeal haemorrhages. Histologically the abomasum showed multifocal to coalescing, severe, ulcerative mucosal lesions with extensive necrosis, mixed with haemorrhage, fibrin, degenerated neutrophils, plasma cells, lymphocytes, and multifocal presence of fungal organisms surrounded by large amounts of fibroblasts. Necrosis and inflammation invaded multifocally into the submucosa. Thrombi were present in multiple vessels in the necrotic areas and the submucosa (Fig. 1B.).

The third calf (Calf 3) showed an abomasal pH of 6 (pH indicator paper, Carl Roth GmbH, Karslruhe (DE)) and was moderately filled with straw and milk. There was a diffuse mild abomasal wall oedema without gross erosions or ulcerations. The rumen, reticulum and omasum appeared normal. Other macroscopic lesions were dehydration, low body condition score (3/9), hepatic pallor and multifocal mild pulmonary and tracheal haemorrhages. Histology was complicated due to moderate post mortem decay. Multifocal discrete abomasal mucosal haemorrhage was seen, without presence of erosions or ulcerations. Also, focal extensive haemorrhages and oedema in the submucosa with focal vasculitis were present (Fig. 1C.).

After clinical examination and confirmation of the initial diagnosis of abomasitis by necropsy, further questioning of the farmer revealed the accidental overdosing of halofuginone lactate. Therefore, the farmer was advised to strictly follow the recommendations of the Halocur® leaflet to prevent further cases. This resulted in no new cases of abomasitis being reported.

## Discussion and conclusions

As far as the authors are aware, no field reports are available of clinical abomasitis associated to halofuginone lactate intoxication. Although, gastro-intestinal inflammatory and necrotic lesions have been reported in calves at 1–3 times the recommended dose in experimental tolerance studies, it is unknown if these resulted in clinical signs. Death of the animals was only reported at 15-fold the recommended dose, which is much higher than what was observed in this case report [7]. The weight loss and hypoproteinaemia were regarded as a consequence of the long period of anorexia.

For in vivo diagnosis of abomasitis gastroscopy is the method of choice, but this is anatomically not possible in ruminants. Hence, ultrasound was used as an alternative non-invasive method. Thickening and edema of the abomasal wall was seen on ultrasonography, and conformed as abomasitis on necropsy. This emphasis the diagnostic value of ultrasound for the diagnosis of abomasitis, although it can only detect severe cases and conditions resulting in edema.

Clostridial abomasitis is the most documented form of abomasitis in calves and also presents with marked edema of the abomasal folds.

The causal link between the high dose of halofuginone lactate and the abomasitis was based on improvement after terminating the administration, and exclusion of other possible causes such as feeding of hyperosmolar solutions, clostridiosis, mechanical irritation, and prolonged usage of non-steroidal anti-inflammatory drugs [13–16]. High osmolality (> 600 mOsm/kg) decreased abomasal emptying rate and resulted in oedema of the abomasal wall [17]. However, even though the osmolality of the milk replacer (382 mOsm/kg) fed on farm was higher than whole milk (300 mOsm/kg), this was not of the magnitude that it would have resulted in substantial decreased abomasal emptying. The lack of abomasal tympany was contraindicative for clostridial involvement. Additionally, one would expect macro- and microscopic signs of a haemorrhagic to necrotizing inflammation of the abomasal wall at necropsy [15]. No samples were taken for bacterial culture, as *Clostridium perfringens* has been isolated from healthy abomasa as well [18]. Furthermore, mechanical irritation was ruled out, due to the absence of abrading structures on ultrasound and necropsy, e.g. trichobezoars.

The underlying mechanisms of the halofuginone lactate administration resulting in the abomasal lesions are still unclear, as the farmer made multiple deviations from the recommendations. He highly overdosed, prolonged administration and administered together with the milk, where post-feeding is recommended [7].

A direct chemical irritation is possible, due to the severely acid properties of halofuginone lactate solution (pH = 2–3, EMA, 2001). Although, no lesions were present in the oesophagus. Therefore, the necessity for a longer contact time, as in the abomasum, in order to establish any caustic effect would explain the regional limitation of the mucosal damage [17]. Alternatively, the abomasitis could be an effect of the decrease in luminal pH due to anorexia, as abomasal hyperacidity is considered to play an important role in the pathogenesis of abomasal ulceration [19]. Nevertheless, the complete mechanisms resulting in the abomasal lesions remains unclear and other causes can not be fully excluded, as straw could also result in mechanical irritation of the abomasum. Although, the amount found on necropsy was low in Calf 6. More research is needed in order to clarify the effect of overdosing of halofuginone lactate in calves and underlying pathogenesis.

The established treatment gave clinical and ultrasonographic cure in four animals. As hyperacidity is linked to long periods of anorexia, proton-pump inhibitors were administered to all calves [16]. This resulted in ultrasonographic cure of the abomasitis in the surviving animals. Although, it could not be stated if similar cure rates would be reached with sole termination of halofuginone lactate administration.

The metaphylactic use of broad-spectrum antibiotics was justified in the authors opinion, due to the high prevalence of possible infectious comorbidities (pneumonia), as well as the poor general condition, and likely immunosuppressive state, that the animals were in [20]. The latter was indicative for a possible immunodeficiency among the presented calves.

This manuscript represents the first suspicion of abomasitis with different clinical lesions associated with overdosing of halofuginone lactate (348–421 µg/kg/day). The animals all showed anorexia, dehydration and weight loss. A key element within the diagnosis was the observation of oedema of the abomasal wall and folds with ultrasound. Removal of halofuginone lactate administration and supportive therapy with treatment of present comorbidities were able to provide clinical recovery in four out of seven animals. More research is needed in order to identify the underlying pathogenesis and treatment possibilities.

## Data Availability

The datasets used and/or analysed during the current study are available from the corresponding author on reasonable request.
